# Intestinal Microbes of Hooded Cranes (*Grus monacha*) Wintering in Three Lakes of the Middle and Lower Yangtze River Floodplain

**DOI:** 10.3390/ani11051390

**Published:** 2021-05-13

**Authors:** Jingjing Gu, Lizhi Zhou

**Affiliations:** 1 School of Resources and Environmental Engineering, Anhui University, Hefei 230601, China; x18301087@stu.ahu.edu.cn; 2 Anhui Province Key Laboratory of Wetland Ecological Protection and Restoration, Anhui University, Hefei 230601, China

**Keywords:** intestinal microbe, Hooded Crane, habitat size, pathogen, wintering waterbirds

## Abstract

**Simple Summary:**

Intestinal microbes are critical to host health, and are affected by environmental factors. In this study, we investigated the intestinal microbes of Hooded Cranes wintering at three lakes with different environmental characteristics in the middle and lower Yangtze River floodplain in China, aiming to provide insights into the effects of habitat size and protection status of birds on their intestinal microbes. We found that the Hooded Cranes at the smaller lake had higher intestinal bacterial and fungal diversity than those at the larger lake. In addition, more diverse and abundant pathogens were found in the gut of Hooded Cranes that lived in the relatively poorly protected habitat than those that lived in well-protected habitat. This study contributes a new perspective for understanding the intestinal microbes of wintering migratory waterbirds at different habitats, and will help to understand the survival status of the vulnerable waterbirds at different habitats for their better conservation.

**Abstract:**

Intestinal microbes participate in life activities of the host, and are affected by external environmental factors. Different habitat sizes and protection status provide different external environmental selection pressures for the same wintering waterbirds, which may be reflected in their intestinal microbes. Hooded Cranes are vulnerable migratory waterbirds with similar numbers wintering at three different lakes in the middle and lower Yangtze River floodplain, Poyang, Caizi, and Shengjin Lakes. Here, we analyzed the characteristics of intestinal bacterial and fungal communities of Hooded Cranes wintering at the three lakes to clarify the effect of habitat size and protection status on intestinal microbes, using high-throughput sequencing technology. Our results showed that community composition and diversity of intestinal microbes were significantly different among lakes with different habitat size and protection status. The Hooded Cranes at Shengjin Lake (small) had higher intestinal microbial alpha-diversity (for both bacteria and fungi) than those at Poyang Lake (large), which might be induced by social behavior of more waterbirds per unit area. The Hooded Cranes at Caizi Lake (relatively poorly protected habitat) had more diverse and abundant intestinal potential pathogens than Shengjin Lake (well-protected habitat). Our results indicated that the environmental pressure of a habitat might affect intestinal microorganisms and more attention might be needed for the vulnerable waterbirds at the habitat of poor protection status.

## 1. Introduction

Birds live in complex and dynamic associations with the symbiotic microbial community in their intestine [1,2]. Intestinal microbes are critical to the health of avian hosts, participating in nutrient absorption and affecting their immunity and metabolism [2,3,4]. The initial intestinal microbes of mammals are acquired during birth and the microbiota is mainly transmitted from the matrix to offspring (vertical transmission) [5]. However, intestinal microbiota is a plastic entity, which can be reconfigured after birth according to different environmental factors (horizontal transmission), and the fluctuation of these microbial communities enables the host to quickly adjust its metabolic and immune performance to cope with environmental changes [6]. Overall, studies have shown that the gut microbiota of avian hosts is largely shaped by the host species [7]; however, environmental factors, as an important driving factor, can also play a crucial role in shaping the characteristics of intestinal microbes [8].

At present, studies on the effects of environmental variables on intestinal microorganisms first are diet [9]. However, other environmental factors, such as social behavior (including interspecific cross-transmission through physical contact due to social crowding), may also alter the intestinal microbiota [10]. For example, a study indicated that direct or indirect contact between the Hooded Cranes and the sympatric Bean Geese led to cross species transmission of intestinal microbes [11]. Moreover, habitat disturbance by humans is considered to be another factor affecting gut microbiota [12]. For example, a study showed that there were significant differences in intestinal microflora between Swan Geese living in the breeding area with limited human disturbance and those living in the wintering area with marked human disturbance [13].

The Hooded Crane (*Grus monacha*) is a typical large East Asian migratory waterbird, breeding mainly in Siberia, Russia, and wintering in Japan, South Korea, and the middle and lower Yangtze River floodplain in China [14]. It has been listed as a vulnerable (VU) species by the International Union for Conservation of Nature (IUCN) and is a national first-class key protected wild animal in China. Hooded Cranes usually forage in clusters within family units, and their wintering habitats are relatively fixed [15]. After arriving at the wintering site from the breeding site, Hooded Cranes mix-forage with other waterbirds and spend nearly 6 months (from October to April) there [16]. Because of the degeneration of natural wetlands and habitat loss, there are only approximately 1050–1150 individuals that winter in China [15]. Poyang, Shengjin and Caizi Lakes are three lakes in the middle and lower Yangtze River floodplain with similar climatic conditions, serving as the main wintering grounds for Hooded Cranes in China [16]. The population numbers of the cranes wintering in these three lakes are very similar, with approximately 300 individuals in each lake [15,17,18].

Here, we used high-throughput sequencing (Illumina MiSeq) to study the intestinal bacteria and fungi community of Hooded Cranes wintering at the three lakes. Poyang and Shengjin Lakes are both National Nature Reserves with similar protection status and similar human disturbances but notably different in size. Different sized lakes have different environmental characteristics, including holding different numbers of migratory waterbirds species and individuals. As interspecific transmission is a potential factor affecting intestinal microbes [11], this implies that when the same migratory waterbirds winter at lakes of different sizes, they may be affected by varying degrees of interspecific intestinal microbial cross-transmission, resulting in differences in their intestinal microbial diversity. Caizi and Shengjin Lakes are similar in size but different in protection status. Caizi Lake is partially a national wetland park and partially a provincial nature reserve. Caizi Lake has been faced with human disturbance since 1950s, including reclaiming land from lake and crab culture in purse seine. This has led to natural habitat degradation, owing to the relatively lower degree of management and protection [19]. The two lake groups have relative different environmental characteristics. Thus, to understand their influence on intestinal microbes, we (i) studied the composition and diversity of intestinal bacterial and fungal communities in Hooded Cranes at the different lake groups (Poyang vs. Shengjin Lake group and Shengjin vs. Caizi Lake group); and (ii) clarified the impact of habitat size and protection status on intestinal microbes.

## 2. Materials and Methods

### 2.1. Ethics Statement

The collection of fecal samples did not involve human interference with the birds, catching, or hunting. Approval was obtained from the local wildlife protection departments. The research process complies with Chinese current laws.

### 2.2. Site Selection and Fecal Sample Collection

**Figure 1:**
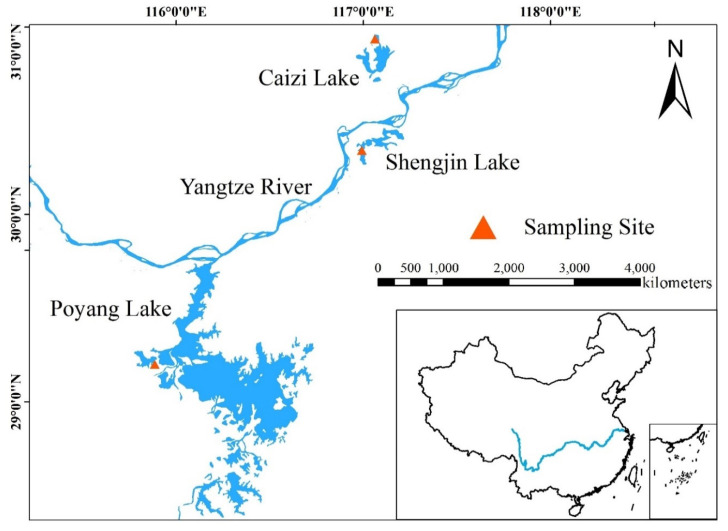
Fecal sampling sites of Hooded Cranes at Poyang, Caizi, and Shengjin Lakes.

The fecal sampling sites were composed of three different sized lakes, fostering most Hooded Crane populations wintering in China. The three lakes were Poyang Lake (28.24°–29.46° N, 115.49°–116.46° E) in Jiangxi Province, Caizi Lake (30.43°–30.58° N, 117.01°–117.09° E) and Shengjin Lake (30.15°–30.28° N, 116.58°–117.14° E) in Anhui Province. Among the three lakes, the maximum surface area of Poyang Lake is more than 4000 km^2^ in wet season and less than 1000 km^2^ in dry season with an average annual water level of 13.88 m [20]. The maximum surface area of Caizi Lake is 226 km^2^ when water level peaks at 14.88 m and 114.97 km^2^ when water level drops to 10.63 m [21]. The maximum surface area of Shengjin Lake ranges from 140 km^2^ in wet season to 75 km^2^ in the dry winter [18]. In this study, the difference between the maximum and minimum water surface areas (maximum surface area minus minimum) was taken as the lake habitat size. The habitat size of Poyang, Caizi, and Shengjin Lakes was more than 3000 km^2^, 111.03 km^2^ and 65 km^2^, respectively. The regions where the lakes are located belong to the northern subtropical monsoon climate, with average annual temperature and precipitation of 14–16 °C and 1000–4000 mm, respectively. All the lakes are river-connected shallow with grass lands, mud flats, and paddy fields, serving as wintering and stopover sites for migratory birds on the East Asian-Australian flyway (Figure 1) [22]. The Hooded Cranes at Poyang and Shengjin Lakes mainly feed on *Polygonum criopolitanum* [23,24], while those at Caizi Lake mainly feed on *Oryza sativa* [25].

Before sampling, we investigated the specific feeding sites of the Hooded Cranes in the three lakes. Then a telescope was used to search for separate Hooded Crane flocks to ensure that there were no other species. Fresh fecal samples were collected immediately after the cranes foraged. Fresh disposable sterile gloves were used to avoid sample contamination. Fecal samples were collected at more than 5 m intervals to avoid collecting samples from the same individual [26]. All the samples were immediately placed in dry ice in a pre-sterilized cooler after collection, transported to the laboratory, and stored at −80 °C before analysis. When conducting formal experiments, the inner core of the feces was used to avoid possible contamination from the outside.

To control for the influence of sampling time on the experiment, sample collection took place roughly at the same time in the three lakes, during 1 March 2019 to 7 March 2019. At this point, the Hooded Cranes have almost completed the whole wintering phases and are ready to leave for the breeding site. Specifically, we collected 71 fecal samples of Hooded Cranes from the three lakes without hurting the cranes. Twenty-seven samples were collected from Poyang Lake (time: 1 March 2019; habitat: grass lands), 22 from Caizi Lake (time: 7 March 2019; habitat: paddy fields), and 22 from Shengjin Lake (time: 7 March 2019; habitat: grass lands).

### 2.3. Fecal DNA Extraction and Bird Species Determination

DNA extraction was performed using the QIAampR DNA Stool Mini Kit (Qiagen, Germany), and quantified by Nano Drop ND-1000 (Thermo Scientific, Waltham, MA, USA). The extracted DNA was then dissolved in elution buffer (60 μL) and stored at −20 °C.

The BIRDF1-BIRDR1 [27] primers were used to amplify the COІ (Cytochrome Oxidase І) gene for identification of the bird species. PCR (Polymerase Chain Reaction) was conducted in a 50μL reaction system consisting of 100 ng fecal DNA from 1 μL DNA template (>20 ng/μL), 22 μL deionized water, 25 μL of 2× EasyTaq PCR Super Mix, 1 μL of the forward primer BIRDF1, and 1 μL of the reverse primer BIRDR1, both at a concentration of 0.2 mM. The specific operation was performed under the following conditions: 5 min of pre-degradation at 95 °C, 30 s of degeneration at 95 °C, 45 s of annealing at 55 °C, 90 s of extension at 72 °C, 35 cycles of repetition of the above process, and a final extension step at 72 °C for 10 min. The PCR results were sent to Sangon Biotech Company for sequencing and BLAST (the Basic Local Alignment Search Tool) analysis (>97% sequence similarity) in NCBI. After confirming that they were from Hooded Cranes, the DNA samples were sent for high-throughput sequencing using an Illumina MiSeq platform.

### 2.4. PCR and Amplicon Library Preparation

The specific process of PCR and amplicon library preparation are described in File S1 (Supplementary Materials) [28,29,30].

### 2.5. Sequence Analysis

The raw sequencing data were preprocessed using the Quantitative Insights into Microbial Ecology (QIIME v.2.0) [31]. Low-quality sequences were removed from our dataset using the deblur algorithm [32]. The remaining high-quality sequences were assigned to amplicon sequence variants (ASVs) based on 100 identity thresholds. The method of VSEARCH was performed to discard the chimers. Then, UNITE database was used to annotate taxonomy to each ASV. Finally, downstream analysis was used to remove the singletons from our dataset.

### 2.6. Statistical Analysis

The difference between intestinal bacteria and fungi among different lake groups was identified by linear discriminant analysis effect size (LEfSe), using nonparametric Kruskal-Wallis rank sum test with the default setting (alpha value: 0.05; effect size threshold: 3.5) [33]. The difference in intestinal microbial community compositions among different groups was analyzed by non-metric multidimensional scaling (NMDS) and analysis of similarity (ANOSIM; permutations = 999), using the vegan package in R (v.3.4.4) [34]. Indicator taxa in different groups were identified by indicator analysis, using the Labdsv package in R. The normal distribution of all data was examined by the Kolmogorov–Smirnov test (*p* > 0.05: normal; *p* ≤ 0.05: non-normal) in SPSS statistics 20.0. One-way analysis of variance (ANOVA) by Tukey honestly significant difference (HSD) comparison was used for the data that followed normal distribution, and Mann-Whitney-Wilcoxon test was used for the data that followed non-normal distribution (Table S1, Supplementary Materials). The raw data were deposited in the National Center for Biotechnology Information (NCBI) Sequence Read Archive (accession number SRP290730 and SRP307396).

## 3. Results

### 3.1. Intestinal Bacterial and Fungal Alpha-Diversity

All the 71 fecal samples were confirmed from the Hooded Cranes after being confirmed by bird species determination (>97% similarity). In this study, 51 samples were selected for analysis according to the ranking of similarity and sequencing quality. Twenty-two samples were from Poyang Lake, 14 from Caizi Lake, and 15 from Shengjin Lake. A total of 800,744 quality-filtered bacterial sequences and 3,164,825 quality-filtered fungal sequences were acquired from the fecal samples of Hooded Cranes at Poyang, Caizi, and Shengjin Lakes. The sequences were grouped into 1526 bacterial and 1282 fungal ASVs which were based on 100% similarity. For intestinal bacteria, the proportion of the ASVs shared by the Hooded Cranes at Poyang and Shengjin Lakes was 21.62%, while the proportion of ASVs shared by the Hooded Cranes at Shengjin and Caizi Lakes was 15.57%. (Figure S1, Supplementary Materials). For intestinal fungi, the proportion of fungal ASVs shared by the Hooded Cranes at Poyang and Shengjin Lakes was 16.28%. The proportion of the fungal ASVs shared by the Hooded Cranes at Shengjin and Caizi Lakes was 23.57% (Figure S1, Supplementary Materials).

**Figure 2:**
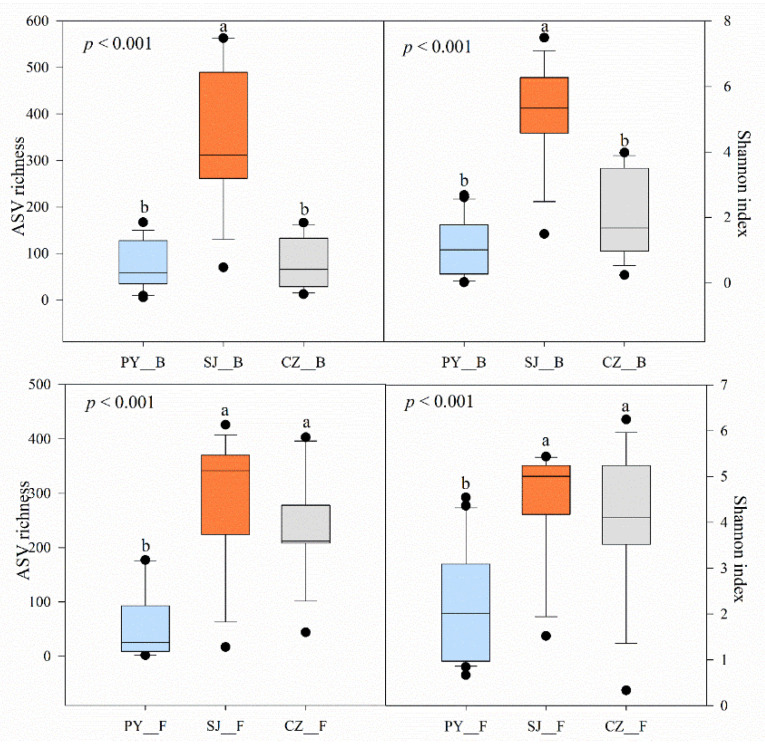
Intestinal microbial alpha-diversity of Hooded Cranes at the three lakes denoted by ASV richness and Shannon index. The panels displayed the interquartile range box plots (first and third quartiles) and whisker (dots indicate manes and the lines inside the box show the medians). Different letters above the boxes indicate highly significant differences according to Tukey HSD test (at *p* < 0.01 level). The same letters above the boxes represent no significant difference (at *p* > 0.05 level). PY: Poyang Lake; CZ: Caizi Lake; SJ: Shengjin Lake; B: bacteria; F: fungi.

The alpha-diversity of the intestinal bacteria and fungi of the Hooded Cranes at Shengjin Lake was significantly higher than that at Poyang Lake (Figure 2). The intestinal bacterial diversity of the Hooded Cranes at Shengjin Lake was significantly higher than those of at Caizi Lake, while the intestinal fungal diversity showed no significant differences with Caizi Lake.

### 3.2. Intestinal Bacterial and Fungal Community Structure

In this study, the intestinal bacteria of the Hooded Cranes at the three lakes were composed of 17 phyla (ranging from 9 to 16 across the three lake samples). The intestinal bacteria phyla of the Hooded Cranes at Poyang and Shengjin Lakes were both dominated by Firmicutes, Proteobacteria, Actinobacteria, and Bacteroidetes, accounting for 98.01% and 95.05% of the total, respectively. The dominant intestinal bacterial phyla of the Hooded Cranes at Caizi Lake were Firmicutes, Proteobacteria, Actinobacteria, accounting for 99.38% of the total. The remaining intestinal bacteria phyla (i.e., Planctomycetes, Fusobacteria etc.) of Hooded Cranes at the three lakes were those with a relative abundance of less than 1%. Except for Bacteroidetes, the distribution of dominant intestinal bacterial phyla among the different lake groups was uneven, as indicated in Table 1. The intestinal bacteria of the Hooded Cranes at the three lakes were composed of 384 genera (ranging from 158 to 321 across the three lake samples). The number of the known bacterial genera (the relative abundance > 0.01%) shared by the Hooded Cranes at Poyang and Shengjin Lake were 52, and the number of the known genera (the relative abundance > 0.01%) shared by the Hooded Cranes at Shengjin and Caizi Lake was 36 (Table S2, Supplementary Materials).

The intestinal fungi of the Hooded Cranes at the three lakes were composed of 11 phyla (ranging from 7 to 9 across the three lake samples). The intestinal fungal phyla of the Hooded Cranes at Poyang and Shengjin Lakes were dominated by Ascomycota, Basidiomycota, Mortierellomycota, and other phyla, accounting for 74.64% and 97.97% of the total, respectively. The intestinal fungal phyla of the Hooded Cranes at Caizi Lake were Ascomycota, Basidiomycota, and other phyla, accounting for 98.15% of the total. The remaining intestinal fungal phyla (i.e., Rozellomycota, Chytridiomycota etc.) of Hooded Cranes at the three lakes were those with a relative abundance of less than 1%. The three known phyla also showed obvious differentiation in different lake groups (Table 1). The intestinal fungi of the Hooded Cranes at the three lakes were composed of 268 genera (ranging from 124 to 212 across the three lake samples). The number of the known fungal genera (relative abundance > 0.01%) shared by the Hooded Cranes at Poyang and Shengjin Lakes was 32, and the number of the known genera (relative abundance > 0.01%) shared by the Hooded Cranes at Shengjin and Caizi Lakes was 44 (Table S3, Supplementary Materials).

**Figure 3:**
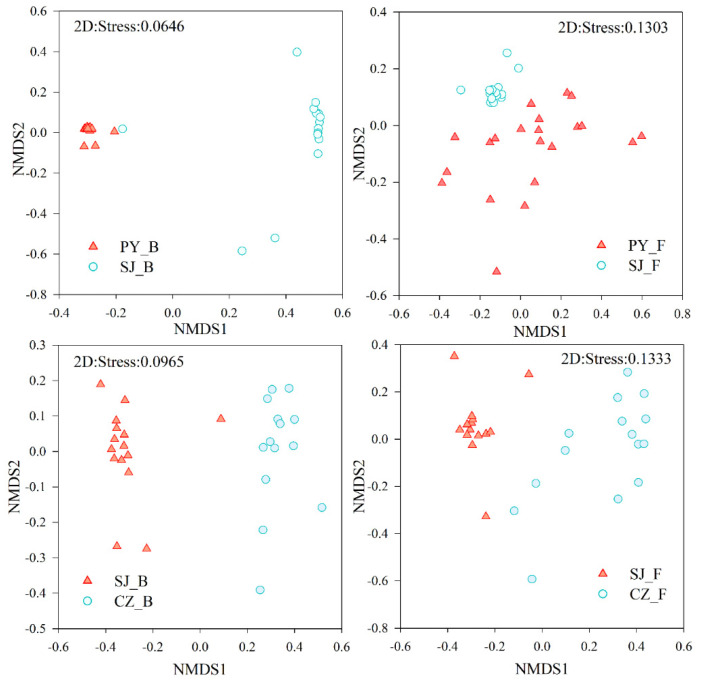
Difference of intestinal bacterial and fungal community composition among different lake groups by NMDS.

Intestinal bacterial and fungal community composition of Hooded Cranes among the different lake groups was identified and compared by NMDS and ANOSIM analysis. The results showed a significant separation between different groups for both bacterial and fungal communities, as indicated in Figure 3 and Table 2 (ANOSIM; *p* < 0.001). However, the intestinal bacterial community composition showed more obvious divergence than the fungal community composition. In addition, ANOSIM indicated that the intestinal fungal community composition of Hooded Cranes at Poyang and Shengjin Lakes (R = 0.2110) was more similar than those at Caizi and Shengjin Lakes (R = 0.7160), though only a little difference was found in the intestinal bacterial community composition between the two lake groups.

### 3.3. Habitat-Specific Bacterial and Fungal Microbiota

**Figure 4:**
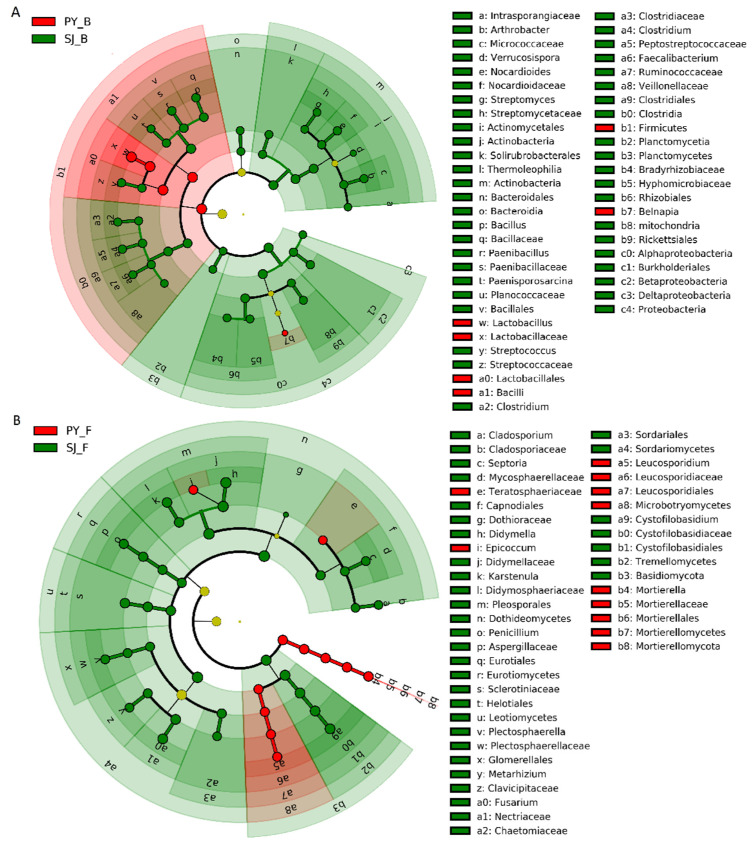
LEfSe analysis of the intestinal bacterial biomarkers (**A**) and fungal biomarkers (**B**) of Hood cranes at Poyang and Shengjin Lake group. Identified phylotype biomarkers were ranked by effect size and the alpha value was < 0.05. Each filled circle represents one biomarker. Cladogram represents the taxonomic hierarchical structure from phylum to genus of the biomarkers identified; Red, phylotypes statistically overrepresented in gut of Hooded Cranes at Poyang Lake; Green, phylotypes statistically overrepresented in gut of Hooded Cranes at Shengjin Lake; Yellow, phylotypes for which relative abundance is not significantly different among three lakes. PY: Poyang Lake; SJ: Shengjin Lake; B: bacteria; F: fungi.

LEfSe analysis revealed specific intestinal bacterial and fungal taxa that differed between the Hooded Cranes at different lake groups. For Poyang and Shengjin Lake group, there were 1 phylum, 1 class, 1 order, 1 family, and 2 genera of intestinal bacteria significantly abundant in the samples from Poyang Lake, while 3 phyla, 8 classes, 8 orders, 15 families and 11 genera of intestinal bacteria were significantly abundant in the samples from Shengjin Lake (Figure 4A). Furthermore, the Shengjin Lake samples also harbored more differential of intestinal fungal taxa than Poyang Lake samples. There were 1 phylum, 5 classes, 7 orders, 12 families, and 9 genera of intestinal fungi significantly abundant in the samples from Shengjin Lake, while 1 phylum, 2 classes, 2 orders, 3 families and 3 genera of intestinal fungi were significantly abundant in the samples from Poyang Lake (Figure 4B).

**Figure 5:**
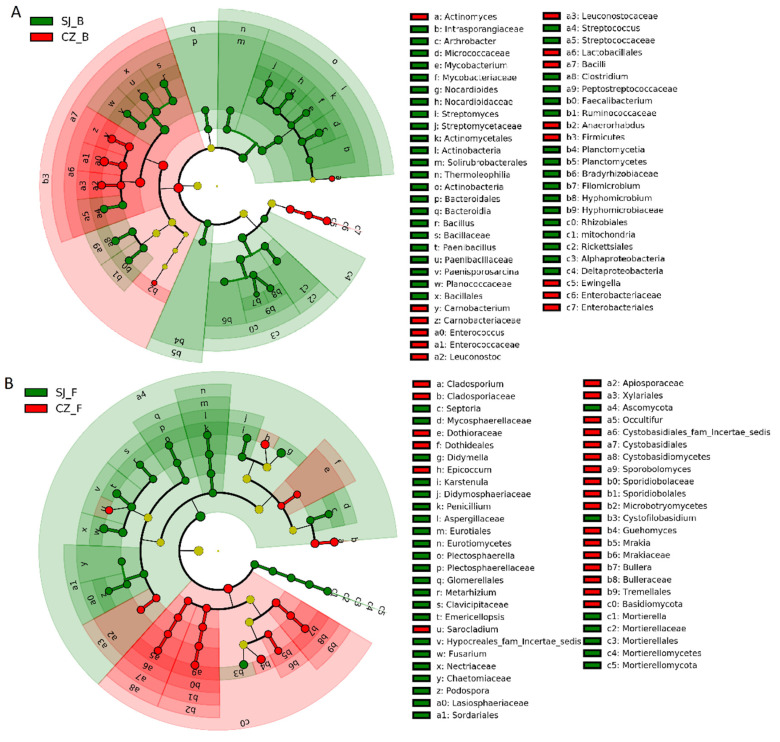
LEfSe analysis of the intestinal bacterial biomarkers (**A**) and fungal biomarkers (**B**) of Hood cranes at Caizi and Shengjin Lake group. Identified phylotype biomarkers were ranked by effect size and the alpha value was < 0.05. Each filled circle represents one biomarker. Cladogram represents the taxonomic hierarchical structure from phylum to genus of the biomarkers identified; Red, phylotypes statistically overrepresented in gut of Hooded Cranes at Caizi Lake; Green, phylotypes statistically overrepresented in gut of Hooded Cranes at Shengjin Lake; Yellow, phylotypes for which relative abundance is not significantly different among three lakes. CZ: Caizi Lake; SJ: Shengjin Lake; B: bacteria; F: fungi.

For Caizi and Shengjin Lake group, there were 1 phylum, 1 class, 2 orders, 4 families, and 6 genera of intestinal bacteria significantly abundant in the samples from Caizi Lake, whereas for Shengjin Lake samples, 2 phyla, 6 classes, 6 orders, and 14 families and 12 genera were significantly abundant (Figure 5A). Furthermore, there were 2 phyla, 2 classes, 5 orders, 7 families, and 8 genera of intestinal fungi significantly abundant at the Caizi Lake samples, while 2 phyla, 2 classes, 4 orders, 10 families and 11 genera of intestinal fungi were significantly abundant in the samples of Shengjin Lake (Figure 5B).

Indicator analysis was used to identify the indicator taxa with respect to each evaluated group, which was conducted at the consensus genera and species level ASVs. If the indicator value is close to 1, which means the indicator taxonomy is a good indicator of the intestinal microbes of Hooded Cranes at the involved lake. However, if the indicator value is close to 0, it means that it is a poor indicator for the lake.

For Poyang and Shengjin Lake group, this analysis revealed 10 ASVs of Hooded Cranes at Shengjin Lake. However, only 1 ASV of Hooded Cranes at Poyang Lake was found as the rest of 9 ASVs with indicator value under 0.6. The only indicator taxa that had a high indicator value (>0.6) of Hooded Cranes at Poyang Lake was ASV372 *Lactobacillus acidipiscis* (51.87%). The indicator taxa of Hooded Cranes at Shengjin Lake were composed of 2 classes, 1 family, 2 genera and 4 species (Table 3).

For Shengjin and Caizi Lake group, a total of 10 ASVs of Hooded Cranes at Shengjin Lake and 10 ASVs of those at Caizi Lake were revealed. The indicator taxa of Hooded Cranes at Shengjin Lake consisted of 2 genera and 6 species. The indicator taxa of Hooded Cranes at Caizi Lake were composed of 2 families, 3 genera and 5 species (Table 4).

## 4. Discussion

Intestinal microbes are thought to be affected by the habitat of host. However, due to the complexity of the habitat, there are few studies on how the habitat affects intestinal microbes. This study investigated the effects of habitat size and protection status on the intestinal microbes of Hooded Cranes.

Divergence in intestinal microbial community composition and alpha-diversity was found between the Hooded Cranes at two different size lakes with a similar habitat protection status (Figure 2 and Figure 3). The Hooded Cranes at the small lake, Shengjin Lake, had significantly higher alpha-diversity of both intestinal bacteria and fungi than those at the large lake, Poyang Lake. Poyang Lake fosters a higher number of waterbird species and individuals (approximately 87 species and more than 400,000 individuals) than Shengjin Lake (more than 80 species and up to 70,000 individuals) [35,36]. However, we found that the number of waterbird species of per unit area of the two lakes was 0.03 (87/3000) species/km^2^ and 1.23 (80/65) species/km^2^, respectively. In addition, the number of waterbirds per unit area of the two lakes was approximately 133 (400,000/3000) ind./km^2^ and 1056 (70,000/65) ind./km^2^, respectively. Thus, the waterbird diversity (a comprehensive metric considering both species and relative abundance of the hosts) of Shengjin Lake per unit area was higher than that of Poyang Lake. As waterbird is a mobile entity, there could be a higher frequency of encounters and more interactions among the waterbirds at Shengjin Lake. Every year, many geese (e.g., Bean Geese, Greater White-Fronted Geese, and Swan Goose) overwinter at Shengjin and Poyang Lakes [15,37]. Previous studies have shown that there was the sharing intestinal microbes between the Hooded Cranes and sympatric other waterbirds, such as the Greater White-Fronted Goose [38], and a recent study demonstrated that the social behaviors (direct or indirect contact) between the Hooded Cranes and the sympatric Bean Goose caused the intestinal bacteria to spread and increased the diversity of intestinal bacteria for both [11]. The possible routes of cross-transmission included feather contamination, physical contact, involuntary fecal feeding, air, water, soil, or other media [39,40]. In this study, the genus of *Megamonas* and *Turicibacter* which were identified as the dominant genus of several goose were both found in the gut of the Hooded Cranes at the two lakes [7,13] (Table S2, Supplementary Materials). The relative abundance of the two genera in the gut of the Hooded Cranes at Shengjin Lake was higher than that of Poyang Lake, indicating that the interaction of intestinal microbial communities through social behaviors might be crucial for changing the intestinal microbial diversity observed in the Hooded Cranes at Shengjin Lake [41]. Furthermore, the relative abundance of the indicator species (*Lactobacillus acidipiscis*) in the gut of Hooded Cranes at Poyang Lake was significantly higher than that of the Hooded Cranes at Shengjin Lake, which was as high as 51.87% (Table 3). *L. acidipiscis* is a potential probiotic that can enhance digestion and suppress the development of certain disease [42,43]. However, the genus *Streptococcus* and *Fusarium* were more abundant in the gut of the Hooded Cranes at Shengjin Lake (Figure 4), both of which might cause disease in the host [44,45]. One effect related to the ecology of animal diseases is called amplification effect, which refers to when increase the species diversity in a community of potential animal hosts for zoonotic pathogens may increase the spread of pathogens among the hosts and increased risk of disease [46]. An example of disease amplification with increased species diversity was that when interspecific transmission is greater than intra-species transmission, the combination of species is more likely to develop pathogens than any single species combination [47]. The possible potential mechanisms include an increase in the abundance of the vectors of the pathogen (vector increase) and an increase in the frequency of encounters between the primary host individuals (encounter increase) [47]. In this study, the population size of Hooded Cranes wintering at the two lakes was small (both approximately 300 individuals). Thus, the influence of interspecific transmission was considered to be greater than intra-species transmission for the Hooded Cranes at the two lakes, which was in accord with the amplification effect example. According to the amplification effect hypothesis, the higher waterbird diversity per unit area might increase the risk of disease [46], which was consistent with our finding. Previous findings showed that dietary, and other external environment factors are important factor for shaping the gut microbial structures for the same host species [13]; however, the Hooded Cranes at Poyang and Shengjin Lakes both mainly feed on *P. criopolitanum* [23,24], indicating the similar diet at the two lakes. Thus, we considered the diet to be a less important factor for this lake group in our study, suggesting that other external environment factors, such as social behavior could be the main possible influencing factors.

Divergence in intestinal microbiota community composition and intestinal bacterial alpha-diversity were also found between the Hooded Cranes at the two lakes with similar size but different in protection status (Figure 2 and Figure 3). The Hooded Cranes at Shengjin Lake had significantly higher intestinal bacterial alpha-diversity than those at Caizi Lake, which had a relatively poor protection status. In the past 30 years, the development of economic aquaculture at Caizi Lake, such as crab farming, has led to extensive nibbling on the roots and tubers of plants that could serve as the food sources of Hooded Cranes [14]. On the other hand, large-scale reclamation of farmland has degraded the wetland area of Caizi Lake and the Hooded Cranes at Caizi Lake began to mainly eat *O. sativa* at the farmland [25]. On the contrary, in recent years, Shengjin Lake has been implemented many effective wetland protection and management measures, including wetland vegetation restoration and banning of seine fishing [48,49]. Therefore, the wetland of Shengjin Lake has remained well protected, and the food resources of the Hooded Cranes are better guaranteed. A study showed that the diet of Hooded Cranes at Shengjin Lake was more diverse and even than that of those at Caizi Lake [25]. The habitat suitability of Shengjin Lake was better than that of Caizi Lake [50]. A previous study on wild black howler monkeys also showed that in less disturbed and relatively primitive habitats, the more diversified diet of black howler monkeys seemed to promote the acquisition and maintenance of various intestinal microbes, while in suboptimal environments, the relatively simple diet tended to reduce diversity [12]. The possible mechanism was that diverse diets provide diverse feeding niches to support diverse microbial taxa and functional taxa [51]. Therefore, a diverse diet might promote the diversity of intestinal microbes, which was consistent with our results. Moreover, the Hooded Cranes at Shengjin Lake mainly fed on high fiber *P. criopolitanum* in the wild. Compared with *O. sativa*, the nutrient content of wild food resources (such as the roots and leaves of certain plants) is relatively lower [52]. The higher diversity of intestinal bacteria might be beneficial for the Hooded Cranes at Shengjin Lake to obtain nutrients effectively from indigestible and nutrient-limited food [53]. Indeed, our study showed that the Hooded Cranes at Shengjin Lake harbored more abundant genus, such as *Bacillus, Clostridium* and *Paenibacillus* which can metabolize cellulose and improve the degradation of non-starch polysaccharides (Figure 5) [54,55]. In addition, we found that the genera *Anaerorhabdus* and *Ewingella* were more abundant in the gut of the Hooded Cranes at Caizi Lake, which might be detrimental for the host and increase the risk of host infection, respectively (Figure 5) [56,57]. Moreover, many indicators (*Ewingella americana*, *Epicoccum nigrum, Enterococcus cecorum*) with a high indicator value of Hooded Cranes at Caizi Lake were also potential pathogenic microbes (Table 4) [57,58,59]. For example, *E. nigrum* could cause respiratory fungal infections in wild birds, such as owls [58]. *E. cecorum* has also been reported to cause severe diseases (e.g., vertebral osteomyelitis) in birds, such as poultry [59]. Both had a relatively high abundance (3.79% and 1.6%) in the gut of Hooded Cranes at Caizi Lake, indicating the cranes there might be suffering more serious pathogen invasion. Interestingly, a previous study showed that there were more pathogenic bacteria in the intestines of Swan Geese in areas with relatively higher human disturbance [13], which was consistent with our study. Rice paddy fields are often used as poultry farms. In the process of sampling, we found that there were poultry breeding sheds near the fields. Poultry have frequent contact with humans and usually roosted in narrow sheds. They could be carriers of pathogens [60], and their feces can pollute paddy fields and water sources during foraging, which could serve as the source of contact infection with enteric pathogens. A recent study demonstrated that there was cross-transmission of intestinal potential pathogens between Hooded Cranes and domestic poultry, and the closer they are, the easier the intestinal flora spread [61], indicating that the more intestinal potentially pathogenic microbes of Hooded Cranes at Caizi Lake may be related to human activities, such as poultry farming. This implied that more conservation measures might be needed to reduce the impact of human activities on the Hooded Cranes at Caizi Lake.

In addition to the difference in intestinal bacterial and fungal community composition of Hooded Cranes at the different lake groups, clustering of intestinal microbiota depending on the habitats (lakes) was detected (Figure 3), which proved that intestinal microbiota showed habitat-preference, implying that the habitats of the host were an important factor in shaping intestinal microbial communities. Furthermore, we found the intestinal fungal community composition of Hooded Cranes at Poyang and Shengjin Lakes was more similar than that of those at Caizi and Shengjin Lakes since the birds in the former lake group have more similar diets. However, the differences in intestinal bacterial community between the two lake groups were almost the same (Table 2), suggesting that although diet is important, environmental factors play a significant role in shaping the intestinal microbial community. Overall, the divergence degree of intestinal fungal communities of Hooded Cranes at the three lakes was less obvious than that of the bacterial communities (Figure 3; Table 2). This is consistent with the results of recent studies in which the composition of the intestinal fungal community was shown to be more variable and less stable than that of the bacterial community [62]; however, the underlying mechanism needs to be further studied.

## 5. Conclusions

In this study, we demonstrated that intestinal microbial community composition and diversity of the Hooded Cranes from three different lakes of the middle and lower Yangtze River floodplain were significantly different. We found that the Hooded Cranes in the small lake, Shengjin Lake, had higher intestinal microbial diversity and relatively more abundant intestinal potential pathogens than those in the large lake (Poyang Lake), which might be attributed to social behavior of more waterbirds per unit area. Our study showed that the Hooded Cranes at Caizi Lake with relatively poor protection had more diverse and abundant intestinal potential pathogens than those at Shengjin Lake with relatively well protected, indicating that the cranes at the less protected habitats might be suffering more serious pathogen invasion and face greater survival pressure. In addition, intestinal bacterial community composition showed a more marked divergence than intestinal fungi. This study contributes to our understanding of the intestinal microbes of wintering migratory waterbirds in different habitats, and can provide a helpful reference for future studies. However, our study has some limitations. No soil samples were collected as controls, and although the inner core of the feces was used to avoid contamination from the outside, the possibility of environmental contamination cannot be completely removed due to the method by which the samples were collected. In addition, only 51 fecal samples were collected. These limitations need to be further clarified in future research.

## Figures and Tables

**Table 1:** Relative abundance of the dominant intestinal bacterial and fungal phyla of the Hooded Cranes at the three lakes.

	Average Relative Abundance (%)				
Phyla	Bacteria	Significance ^1^	Phyla	Fungi	Significance ^1^
	(PY|SJ|CZ)	(PY-SJ|SJ-CZ)		(PY|SJ|CZ)	(PY-SJ|SJ-CZ)
Firmicutes	88.42|65.08|84.21	(a-b *|b-a)	Ascomycota	57.74|70.80|49.13	(a-a|a-b *)
Proteobacteria	8.36|16.94|11.20	(b-a|a-a)	Basidiomycota	8.03|10.61|27.35	(b-a *|b-a)
Actinobacteria	1.23|13.03|3.97	(b-a *|a-b *)	Mortierellomycota	4.45|2.92|—	(a-b *|—)
Bacteroidetes	1.56|2.5|—	(a-a|—)	others	4.42|13.64|21.67	—

^1^ Different letters indicate significant difference between lakes according to Tukey HSD test or Mann–Whitney test (both at *p* < 0.05 level). “*” represents highly significant difference (*p* < 0.01). The same letters in brackets represent no significant difference (*p* > 0.05). PY: Poyang Lake, SJ: Shengjin Lake, CZ: Caizi Lake.

**Table 2:** Difference of intestinal bacterial and fungal community composition among different lake groups based on ANOSIM.

	Bacteria Variable			Fungi Variable	
	*R*	*p*		*R*	*p*
PY_B vs. SJ_B	0.9325	0.001	PY_F vs. SJ_F	0.2110	0.003
SJ_B vs. CZ_B	0.9231	0.001	SJ_F vs. CZ_F	0.7160	0.001

PY: Poyang Lake; CZ: Caizi Lake; SJ: Shengjin Lake; B: bacteria; F: fungi.

**Table 3:** Intestinal bacterial and fungal indicator species of Hooded Crane of the lake groups between Poyang and Shengjin Lakes. The top five indicator taxa according to the indicated value were listed (except for indicator value < 0.6). o: order; f: family; g: genus. PY: Poyang Lake; SJ: Shengjin Lake; B: bacteria; F: fungus.

Treatment	Indicator Species	Indicator Value	*p*	Relative Abundance (%)	Taxonomy
PY_B	ASV372	0.9530	0.001	51.87	*Lactobacillus acidipiscis*
SJ_B	ASV7	0.9965	0.001	0.47	g__*Paenisporosarcina*
SJ_B	ASV6	0.9902	0.001	0.57	g__*Paenibacillus*
SJ_B	ASV18	0.9888	0.001	0.32	o__*Rhizobiales*
SJ_B	ASV5	0.9872	0.001	1.13	g__*Paenibacillus*
SJ_B	ASV8	0.9791	0.001	0.49	g__*Paenibacillus*
SJ_F	ASV1567	1.0000	0.001	0.43	*Septoria sonchi*
SJ_F	ASV1606	1.0000	0.001	0.23	f__Lasiosphaeriaceae
SJ_F	ASV1565	0.9875	0.001	0.61	*Plectosphaerella cucumerina*
SJ_F	ASV1540	0.9640	0.001	2.74	*Fusarium oxysporum*
SJ_F	ASV1571	0.9454	0.001	0.32	o__Hypocreales

**Table 4:** Intestinal bacterial and fungal indicator species of Hooded Crane of the lake groups between Shengjin and Caizi Lakes. The top five indicator taxa according to the indicated value were listed (except for indicator value < 0.6). f: family g: genus. SJ: Shengjin Lake; CZ: Caizi Lake; B: bacteria; F: fungus.

Treatment	Indicator ASV	Indicator Value	*p*	Relative Abundance (%)	Taxonomy
SJ_B	ASV10	1.000	0.001	0.28	g__*Paenibacillus*
SJ_B	ASV19	1.000	0.001	0.44	g__*Paenibacillus*
SJ_B	ASV22	1.000	0.001	0.29	g__*Paenibacillus*
SJ_B	ASV23	1.000	0.001	0.16	g__*Paenisporosarcina*
SJ_B	ASV52	0.9907	0.001	0.05	*Methylobacterium adhaesivum*
SJ_F	ASV1541	1.000	0.001	0.27	*Emericellopsis humicola*
SJ_F	ASV1567	1.000	0.001	0.53	*Septoria sonchi*
SJ_F	ASV1540	0.9987	0.001	3.22	*Fusarium oxysporum*
SJ_F	ASV1592	0.9927	0.001	0.32	*Emericellopsis humicola*
SJ_F	ASV1565	0.9921	0.001	0.74	*Plectosphaerella cucumerina*
CZ_B	ASV1527	0.9165	0.001	3.47	*Lactobacillus acidipiscis*
CZ_B	ASV1529	0.8559	0.001	1.60	*Enterococcus cecorum*
CZ_B	ASV1581	0.7857	0.001	0.07	g__*Candidatus Arthromitus*
CZ_B	ASV1532	0.7143	0.001	0.72	*Ewingella americana*
CZ_B	ASV1543	0.7143	0.001	0.27	g__*Martelella*
CZ_F	ASV1545	0.9869	0.001	1.80	*Bullera japonica*
CZ_F	ASV1548	0.9715	0.001	1.95	g__*Sporobolomyces*
CZ_F	ASV1531	0.9324	0.001	3.79	*Epicoccum nigrum*
CZ_F	ASV1559	0.9303	0.001	0.62	*Sarocladium strictum*
CZ_F	ASV1553	0.9219	0.002	1.59	f__Sporormiaceae

## Data Availability

The raw data were deposited in the National Center for Biotechnology Information (NCBI) Sequence Read Archive (accession number SRP290730 and SRP307396).

## Supplementary Materials

The following are available online at https://www.mdpi.com/article/10.3390/ani11051390/s1. File S1: Supporting Materials and Methods: PCR and amplicon library preparation, Figure S1: Venn diagram showing the unique and shared intestinal bacterial (A,B) and fungal (C,D) ASVs of Hooded Cranes at different lake groups. PY: Poyang lake; CZ: Caizi Lake; SJ: Shengjin Lake; B: bacteria; F: fungus, Table S1: The distribution of data was analyzed by Kolmogorov-Smirnov test. PY: Poyang Lake; SJ: Shengjin Lake; CZ: Caizi Lake. Normal distribution: *p* > 0.05; Non-normal distribution: *p* ≤ 0.05, Table S2: The relative abundance of the intestinal bacterial genera shared by the Hooded Cranes at different lake groups. The genus with a relative abundance of more than 0.01% are listed. The generic names that are the same are distinguished by family names in the bracket. PY: Poyang Lake; SJ: Shengjin Lake; CZ: Caizi Lake; B: bacteria, Table S3: The relative abundance of the intestinal fungal genera shared by the Hooded Cranes at different lake groups. The genus with a relative abundance of more than 0.01% are listed. PY: Poyang Lake; SJ: Shengjin Lake; CZ: Caizi Lake; F: fungi.

## References

[1] Wang W., Wang A., Yang Y., Wang F., Liu Y., Zhang Y., Sharshov K., Gui L. (2019). Composition, diversity and function of gastrointestinal microbiota in wild red-billed choughs (*Pyrrhocorax pyrrhocorax*). Int. Microbiol..

[2] Cisek A.A., Binek M. (2014). Chicken intestinal microbiota function with a special emphasis on the role of probiotic bacteria. Pol. J. Vet. Sci..

[3] Veelen P., Falcao S.J., Matson K.D., Velde M., Tieleman B.I. (2020). Microbial environment shapes immune function and cloacal microbiota dynamics in Zebra Finches *Taeniopygia guttata*. Anim. Microbiome.

[4] Elokil A.A., Magdy M., Melak S., Ishfaq H., Bhuiyan A., Cui L., Jamil M., Zhao S., Li S. (2020). Faecal microbiome sequences in relation to the egg-laying performance of hens using amplicon-based metagenomic association analysis. Animal.

[5] Leser T.D., Mølbak L. (2009). Better living through microbial action: The benefits of the mammalian gastrointestinal microbiota on the host. Environ. Microbiol..

[6] Candela M., Biagi E., Maccaferri S., Turroni S., Brigidi P. (2012). Intestinal microbiota is a plastic factor responding to environmental changes. Trends Microbiol..

[7] Yang Y., Deng Y., Cao L. (2016). Characterising the interspecific variations and convergence of gut microbiota in Anseriformes herbivores at wintering areas. Sci. Rep..

[8] Grond K., Santo Domingo J.W., Lanctot R.B., Jumpponen A., Bentzen R.L., Boldenow M.L., Brown S.C., Casler B., Cunningham J.A., Doll A.C. (2019). Composition and drivers of gut microbial communities in arctic-breeding shorebirds. Front. Microbiol..

[9] Wu G.D., Chen J., Hoffmann C., Bittinger K., Chen Y.Y., Keilbaugh S.A., Bewtra M., Knights D., Walters W.A., Knight R. (2011). Linking long-term dietary patterns with gut microbial enterotypes. Science.

[10] Blaser M.J., Falkow S. (2019). What are the consequences of the disappearing human microbiota?. Nat. Rev. Microbiol..

[11] Yang Z., Zhou L. (2021). Is intestinal bacterial diversity enhanced by trans-species spread in the mixed-species flock of Hooded Crane (*Grus monacha*) and Bean Geese (*Anser fabalis*) wintering in the Lower and Middle Yangtze River floodplain?. Animals.

[12] Amato K.R., Yeoman C.J., Kent A., Righini N., Carbonero F., Estrada A., Gaskins H.R., Stumpf R.M., Yildirim M., Torralba M. (2013). Habitat degradation impacts black howler monkey (*Alouatta pigra*) gastrointestinal microbiomes. ISME J..

[13] Wu Y., Yang Y., Cao L., Yin H., Xu M., Wang Z., Liu Y., Wang X., Deng Y. (2018). Habitat environments impacted the gut microbiome of long-distance migratory Swan Geese but central species conserved. Sci. Rep..

[14] Zheng M., Zhou L.Z., Zhao N.N., Xu W.B. (2015). Effects of variation in food resources on foraging habitat use by wintering Hooded Cranes (*Grus monacha*). Avian Res..

[15] Zhu Z., Zhou L., Yu C., Cheng L., Xu W., Song Y. (2020). Do geese facilitate or compete with wintering Hooded Cranes (*Grus monacha*) for forage resources?. Diversity.

[16] Huang W., Zhou L., Zhao N. (2014). Temporal-spatial patterns of intestinal parasites of the Hooded Crane (*Grus monacha*) wintering in lakes of the middle and lower Yangtze River floodplain. Avian Res..

[17] Shao M.Q., Guo H., Jiang J.H. (2014). Population sizes and group characteristics of Siberian Crane (*Leucogeranus leucogeranus*) and Hooded Crane (*Grus monacha*) in Poyang Lake Wetland. Zool. Res..

[18] Wei Z., Zheng M., Zhou L., Xu W. (2020). Flexible foraging response of wintering Hooded Cranes (*Grus monacha*) to food availability in the lakes of the Yangtze River floodplain, China. Animals.

[19] Wang X., Chen J., Zhou L. (2020). Effects of human activities on the diversity of waterbirds wintering in a shallow lake of the middle and lower Yangtze River floodplain, China. Diversity.

[20] Teng J., Xia S., Liu Y., Yu X., Duan H., Xiao H., Zhao C. (2021). Assessing habitat suitability for wintering geese by using Normalized Difference Water Index (NDWI) in a large floodplain wetland, China. Ecol. Indic..

[21] Li C., Li H., Zhang Y., Zha D., Zhao B., Yang S., Zhang B., Boer W.F. (2019). Predicting hydrological impacts of the Yangtze-to-Huaihe Water Diversion Project on habitat availability for wintering waterbirds at Caizi Lake. J. Environ. Manag..

[22] Cao L., Fox A.D. (2009). Birds and people both depend on China’s wetlands. Nature.

[23] Hou J. (2019). Diet Niche Partitioning by Four Wintering Cranes in Poyang Lake. Master’s Thesis.

[24] Zhang N., Zhou L., Yang Z., Gu J. (2021). Effects of food changes on intestinal bacterial diversity of wintering Hooded Cranes (*Grus monacha*). Animals.

[25] Zhou L. (2013). Seasonal Shifts of Food Habit of the Hooded Crane (*Grus monacha*) Wintering in the Lakes of Yangtze River Floodplain in Anhui Province. Master’s Thesis.

[26] Zhang F., Xiang X., Dong Y., Yan S., Song Y., Zhou L. (2020). Significant differences in the gut bacterial communities of Hooded Crane (*Grus monacha*) in different seasons at a stopover site on the flyway. Animals.

[27] Hebert P.D., Stoeckle M.Y., Zemlak T.S., Francis C.M. (2004). Identification of Birds through DNA Barcodes. PLoS Biol..

[28] Yusoff M.Z.M., Hu A.Y., Feng C.J., Maeda T., Shirai Y., Hassan M.A., Yu C.P. (2013). Influence of pretreated activated sludge for electricity generation in microbial fuel cell application. Bioresour. Technol..

[29] Lemons A.R., Barnes C.S., Green B.J. (2017). Comparative analysis of Sanger and Illumina Miseq Sequencing for determining indoor fungal diversity. J. Allergy Clin. Immunol..

[30] Caporaso J.G., Lauber C.L., Walters W.A., Berg-Lyons D., Huntley J., Fierer N., Owens S.M., Betley J., Fraser L., Bauer M. (2012). Ultra-high-throughput microbial community analysis on the Illumina HiSeq and MiSeq platforms. ISME J..

[31] Caporaso J.G., Kuczynski J., Stombaugh J., Bittinger K., Bushman F.D., Costello E.K., Fierer N., Pena A.G., Goodrich J.K., Gordon J.I. (2010). QIIME allows analysis of high-throughput community sequencing data. Nat. Methods.

[32] Wang Q., Garrity G.M., Tiedje J.M., Cole J.R. (2007). Naïve Bayesian classifier for rapid assignment of rRNA sequences into the new bacterial taxonomy. Appl. Environ. Microbiol..

[33] Segata N., Izard J., Waldron L., Gevers D., Miropolsky L., Garrett W.S., Huttenhower C. (2011). Metagenomic biomarker discovery and explanation. Genome Biol..

[34] Anderson M.J., Walsh D.C.I. (2013). PERMANOVA, ANOSIM, and the Mantel test in the face of heterogeneous dispersions: What null hypothesis are you testing?. Ecol. Monogr..

[35] Wang W., Fraser J.D., Chen J. (2017). Distribution and long-term population trends of wintering waterbirds in Poyang Lake, China. Wetlands.

[36] Li C., Yang Y., Wang Z., Yang L., Zhang D., Zhou L. (2018). The relationship between seasonal water level fluctuation and habitat availability for wintering waterbirds at Shengjin Lake, China. Bird Conserv. Int..

[37] Li Y., Qian F., Silbernagel J., Larson H. (2019). Community structure, abundance variation and population trends of waterbirds in relation to water level fluctuation in Poyang Lake. J. Great Lakes Res..

[38] Xiang X., Zhang F., Fu R., Yan S., Zhou L. (2019). Significant differences in bacterial and potentially pathogenic communities between sympatric Hooded Crane and Greater White-Fronted Goose. Front. Microbiol..

[39] Hubálek Z. (2004). An annotated checklist of pathogenic microorganisms associated with migratory birds. J. Wildlife Dis..

[40] Alm E.W., Daniels-Witt Q.R., Learman D.R., Ryu H., Jordan D.W., Gehring T.M., Jorge S.D. (2018). Potential for gulls to transport bacteria from human waste sites to beaches. Sci. Total Environ..

[41] Moeller A.H., Foerster S., Wilson M.L., Pusey A.E., Hahn B.H., Ochman H. (2016). Social behavior shapes the chimpanzee pan-microbiome. Sci. Adv..

[42] Altaher Y., Jahromi M., Ebrahim R., Zulkifli I., Liang J. (2015). *Lactobacillus Pentosus* Ita23 and *L. Acidipiscis* Ita44 enhance feed conversion efficiency and beneficial gut microbiota in broiler chickens. Braz. J. Poult. Sci..

[43] Ren S., Zhang X., Guan H., Wu L., Yu M., Hou D., Yan Y., Fang X. (2021). *Lactobacillus acidipiscis* induced regulatory gamma delta T cells and attenuated experimental autoimmune encephalomyelitis. Front. Immunol..

[44] Gomes M., Tako E. (2021). Effects of iron and zinc biofortified foods on gut microbiota in vivo (*Gallus gallus*): A systematic review. Nutrients.

[45] Keçi M., Lucke A., Paulsen P., Zebeli Q., Böhm J., Metzler-Zebeli B.U. (2019). Deoxynivalenol in the diet impairs bone mineralization in broiler chickens. Toxins.

[46] Rubio A.V., Castro-Arellano I., Mills J.N., List R., Avila-Flores R., Suzan G. (2017). Is species richness driving intra- and interspecific interactions and temporal activity overlap of a hantavirus host? An experimental test. PLoS ONE.

[47] Keesing F., Holt R.D., Ostfeld R.S. (2006). Effects of species diversity on disease risk. Ecol. Lett..

[48] Zhou J., Zhou L., Xu W. (2020). Diversity of wintering waterbirds enhanced by restoring aquatic vegetation at Shengjin Lake, China. Sci. Total Environ..

[49] Dibar D.T., Zhang K., Yuan S., Zhang J., Zhong Z., Ye X. (2020). Ecological stoichiometric characteristics of carbon (C), nitrogen (N) and phosphorus (P) in leaf, root, stem, and soil in four wetland plants communities in Shengjin Lake, China. PLoS ONE.

[50] Chen W., Zhou L., Wang W., Song Y., Zhang H. (2020). Habitat suitability of wintering Hooded Crane in Shengjin Lake and Caizi Lake. Wetl. Sci..

[51] Louis P., Scott K.P., Duncan P., Flint H.J. (2007). Understanding the effects of diet on bacterial metabolism in the large intestine. J. Appl. Microbiol..

[52] Xiang X., Jin L., Yang Z., Zhang N.Z., Zhang F.L. (2021). Dramatic shifts in intestinal fungal community between wintering Hooded Crane and Domestic Goose. Avian Res..

[53] Cantarel B.L., Lombard V., Henrissat B. (2012). Complex carbohydrate utilization by the healthy human microbiome. PLoS ONE.

[54] Zhao G., Zhou L., Dong Y., Cheng Y., Song Y. (2017). The gut microbiome of Hooded Cranes (*Grus monacha*) wintering at Shengjin Lake, China. Microbiol. Open.

[55] Xia Y., Kong J., Zhang G., Zhang X.X., Seviour R., Kong Y.H. (2019). Effects of dietary supplementation with lysozyme on the structure and function of the cecal microbiota in broiler chickens. PLoS ONE.

[56] Liu C., Cheng L., Ji L., Li F., Cheng Y. (2020). Intestinal microbiota dysbiosis play a role in pathogenesis of patients with primary immune thrombocytopenia. Thromb. Res..

[57] Khurana S., Chemmachel C., Saxena R. (2020). *Ewingella americana* peritonitis in a patient on peritoneal dialysis: A case report and review of the literature. Case Rep. Nephrol. Dial..

[58] Rhim H., Park J.Y., Lee D.J., Han J.I. (2019). *Epicoccum nigrum*-induced respiratory infection in a wild Eurasian Scops Owl (*Otus scops*). J. Vet. Med. Sci..

[59] Delaunay E., Abat C., Rolain J.M. (2015). *Enterococcus cecorum* human infection, France. New Microbes New Infect..

[60] Fu R., Xiang X., Dong Y., Cheng L., Zhou L. (2020). Comparing the intestinal bacterial communities of sympatric wintering Hooded Crane (*Grus monacha*) and Domestic Goose (*Anser anser domesticus*). Avian Res..

[61] Wang W., Zhou L., Fu R., Cheng L., Yan S., Mahtab N., Song Y. (2021). Effects of foraging site distances on the intestinal bacterial community compositions of the sympatric wintering Hooded Crane (*Grus monacha*) and Domestic Duck (*Anas platyrhynchos domesticus*). Avian Res..

[62] Hallen-Adams H.E., Suhr M.J. (2017). Fungi in the healthy human gastrointestinal tract. Virulence.

