# Supplementary material for: Intestinal Microbes of Hooded Cranes (Grus monacha) Wintering in Three Lakes of the Middle and Lower Yangtze River Floodplain
Source: Animals (Basel). 2021 May 13;11(5):1390. doi: 10.3390/ani11051390 (PMC8153004; doi:10.3390/ani11051390)
Supplement: Supplementary file 1 [file animals-11-01390-s001.zip › animals-1201265-file 1.pdf]

File S1 Supporting Materials and Methods: PCR and amplicon library preparation

Purified DNA (50 μg) was used as amplification template for each sample. F515/R907 was used as bacterial primer to amplify the V4-V5 hypervariable region of the bacterial 16S rRNA gene fragments [28], and ITS1F/ITS2R was used as fungal primer to amplify the ITS1 hypervariable region of fungal ITS gene fragments [29] at the Illumina MiSeq platform in Majorbio (Shanghai, China) [30]. PCR was performed in a 50 μL reaction mixture consisting of each dNTP at a concentration of 200 μM, forward and reverse primers at a concentration of 0.4 μM and 2 U Taq DNA polymerase. Cycling was performed under the following operating conditions: denaturation at 94 °C for 45 s, 35 cycles of annealing at 55 °C for 45 s, extension at 72 °C for 45 s and an elongation step at 72 °C for 10 min. Further, we used PCR negative controls without adding DNA templates to check for contamination. The agarose gel DNA purification kit (TaKaRa, Japan) was used to purify the three reaction mixtures from each sample. Finally, we pooled the PCR product (10 pg) in equimolar quantity to sequence each sample. Sequencing was then performed at the Illumina MiSeq platform.

References

28. Yusoff, M.Z.M.; Hu, A.Y.; Feng, C.J.; Maeda, T.; Shirai, Y.; Hassan, M.A.; Yu, C.P. Influence of pretreated activated sludge for electricity generation in microbial fuel cell application. *Bioresour. Technol.* **2013**, *145*, 90-96, doi:10.1016/j.biortech.2013.03.003.

29. Lemons, A.R.; Barnes, C.S.; Green, B.J. Comparative analysis of Sanger and Illumina Miseq Sequencing for determining indoor fungal diversity. *J. Allergy Clin. Immunol*. **2017**, *139*, 2, AB86, doi:10.1016/j.jaci.2016.12.233.

30. Caporaso, J.G.; Lauber, C.L.; Walters, W.A.; Berg-Lyons, D.; Huntley, J.; Fierer, N.; Owens, S.M.; Betley, J.; Fraser, L.; Bauer, M., et al. Ultra-high-throughput microbial community analysis on the Illumina HiSeq and MiSeq platforms. *ISME J* **2012**, *6*, 1621-1624, doi:10.1038/ismej.2012.8.
