# Supplementary material for: Intestinal Microbes of Hooded Cranes (Grus monacha) Wintering in Three Lakes of the Middle and Lower Yangtze River Floodplain
Source: Animals (Basel). 2021 May 13;11(5):1390. doi: 10.3390/ani11051390 (PMC8153004; doi:10.3390/ani11051390)
Supplement: Supplementary file 1 [file animals-11-01390-s001.zip › animals-1201265-file 2.pdf]

Supplementary Materials:

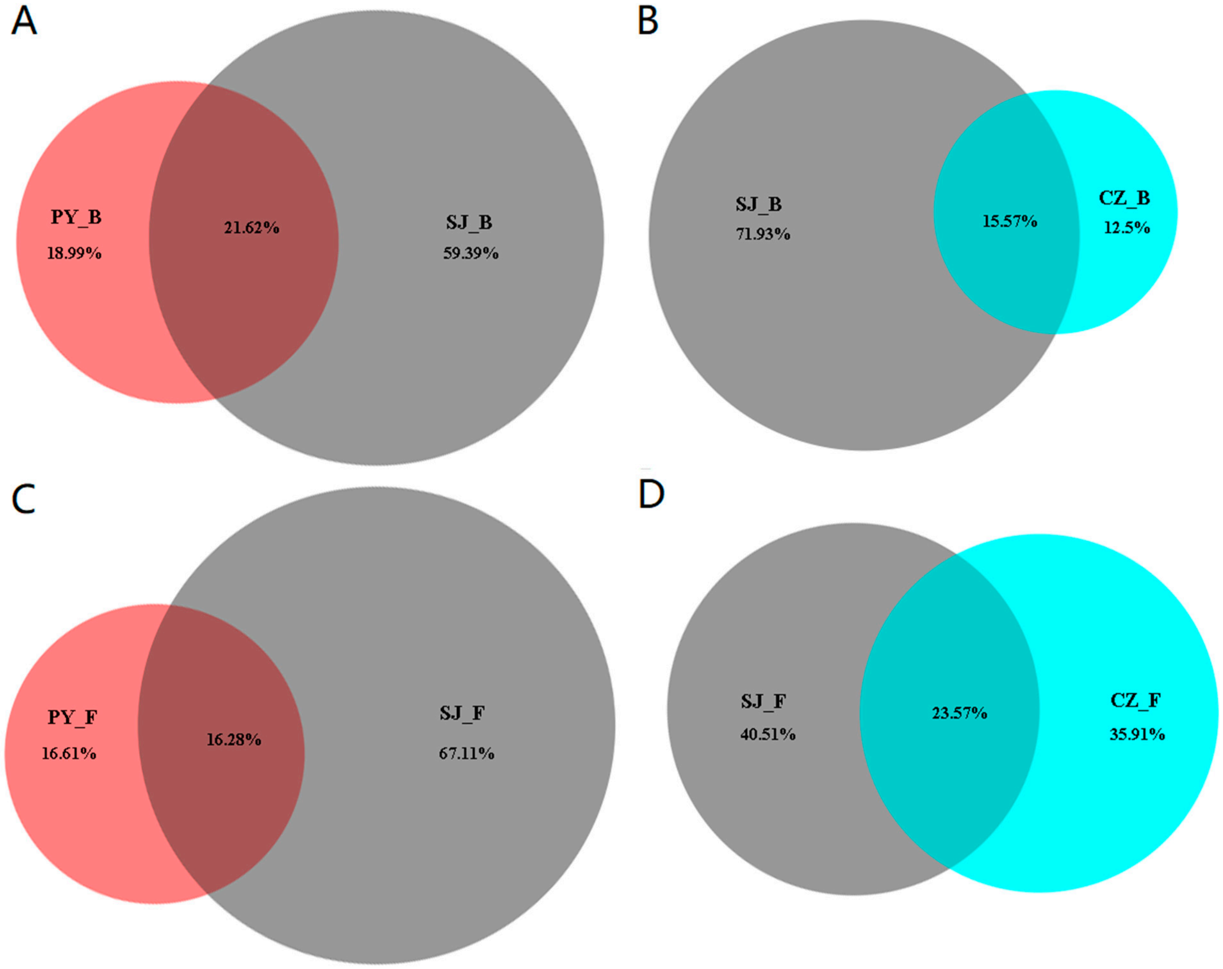

Figure S1. Venn diagram showing the unique and shared intestinal bacterial (**A** and **B**) and fungal (**C** and **D**) ASVs of Hooded Cranes at different lake groups . PY: Poyang lake; CZ: Caizi Lake; SJ: Shengjin Lake; B: bacteria; F: fungus.

Table S1. The distribution of data was analyzed by Kolmogorov-Smirnov test. PY: Poyang Lake; SJ: Shengjin Lake; CZ: Caizi Lake. Normal distribution: $p > 0.05$; Non-normal distribution: $p \leq 0.05$.

Lake group	Treatment	Kolmogorov-Smirnov test (*P* value)	Distribution
PY vs. SJ	Bacterial ASV richness	0.120	Normal
	Bacterial Shannon index	0.324	Normal
	Fungal ASV richness	0.103	Normal
	Fungal Shannon index	0.466	Normal
	Bacterial Firmicutes Phyla	0.321	Normal
	Bacterial Proteobacteria Phyla	0.134	Normal
	Bacterial Actinobacteria Phyla	0.035	Non-normal
	Bacterial Bacteroidetes Phyla	<0.001	Non-normal
	Fungal Ascomycota Phyla	0.052	Normal
	Fungal Basidiomycota Phyla	0.003	Non-normal
	Fungal Mortierellomycota Phyla	<0.001	Non-normal
SJ vs. CZ	Bacterial ASV richness	0.365	Normal
	Bacterial Shannon index	0.738	Normal
	Fungal ASV richness	0.509	Normal
	Fungal Shannon index	0.303	Normal
	Bacterial Firmicutes Phyla	0.876	Normal
	Bacterial Proteobacteria Phyla	0.769	Normal
	Bacterial Actinobacteria Phyla	0.445	Normal
	Bacterial Bacteroidetes Phyla	<0.001	Non-normal
	Fungal Ascomycota Phyla	0.095	Normal
	Fungal Basidiomycota Phyla	0.277	Normal
	Fungal Mortierellomycota Phyla	0.006	Non-normal

Table S2. The relative abundance of the intestinal bacterial genera shared by the Hooded Cranes at different lake groups. The genus with a relative abundance of more than 0.01% are listed. The generic names that are the same are distinguished by family names in the bracket. PY: Poyang Lake; SJ: Shengjin Lake; CZ: Caizi Lake; B: bacteria.

PY_B vs. SJ_B			SJ_B vs. CZ_B		
Genus	Relative abundance (%)		Genus	Relative abundance (%)	
	PY_B	SJ_B		SJ_B	CZ_B
Lactobacillus	85.27	31.18	*Lactobacillus*	31.18	48.61
Pseudomonas	2.53	0.32	*Paenibacillus*	5.87	0.03
Escherichia	2.42	0.32	*Clostridium*	3.16	0.15
Bacillus	0.64	5.70	*Nocardioides*	2.77	0.06
Methylobacterium	0.49	0.84	*Streptococcus*	2.65	0.02
Mycobacterium	0.38	0.82	*Arthrobacter*	2.53	0.11
Martelella	0.33	0.14	*Clostridium*(Clostridiaceae)	1.66	2.57
Clostridium(Clostridiaceae)	0.26	1.66	*Streptomyces*	1.03	0.28
Acinetobacter	0.23	0.08	*Methylobacterium*	0.84	0.40
Streptococcus	0.19	2.65	*Mycobacterium*	0.82	0.08
Campylobacter	0.15	0.13	*Massilia*	0.74	0.02
Clostridium(Peptostreptococcaceae)	0.15	3.16	*Enterococcus*	0.39	19.41
Megamonas	0.15	0.32	*Pseudomonas*	0.32	2.87
Enterococcus	0.14	0.39	*Megamonas*	0.32	0.02
Bacteroides	0.11	0.34	*Rhizobium*	0.31	0.01
Lactococcus	0.10	0.02	*Terracoccus*	0.30	0.01
Paenibacillus	0.10	5.87	*Turicibacter*	0.29	0.02
Nocardioides	0.09	2.77	*BSV43*	0.24	0.02
Solirubrobacter	0.07	0.12	*Pedobacter*	0.18	0.03
Aurantimonas	0.07	0.05	*Rhodococcus*	0.15	0.39
Planctomyces	0.07	0.23	*Martelella*	0.14	1.85
Rhizobium	0.07	0.31	*Pelosinus*	0.14	0.38
Streptomyces	0.06	1.03	*Campylobacter*	0.13	0.24
Rothia	0.06	0.01	*Acidovorax*	0.07	0.18
[Clostridium]	0.06	0.05	*Microbacterium*	0.06	0.25
Microbacterium	0.06	0.06	*Aurantimonas*	0.05	0.06
Clostridium(Lachnospiraceae)	0.05	0.04	*[Clostridium]*	0.05	0.06
Labrys	0.05	0.06	*Actinomycetospora*	0.05	0.01
Faecalibacterium	0.04	1.51	*Clostridium*(Lachnospiraceae)	0.04	0.12
Rhodococcus	0.03	0.15	*Intrasporangium*	0.03	0.01
Nostocoida	0.03	0.12	*Sphingomonas*	0.02	0.01
Hyphomicrobium	0.03	0.62	*Lactococcus*	0.02	0.50
Actinomycetospora	0.03	0.05	*Frigoribacterium*	0.02	0.05
Massilia	0.03	0.74	*Corynebacterium*	0.01	0.02
Afipia	0.03	0.41	*Kaistia*	0.01	0.22

Rhodoblastus	0.03	0.07	*Butyricicoccus*	0.01	0.08
Arthrobacter	0.02	2.53	—	—	—
Candidatus Solibacter	0.02	0.02	—	—	—
Kaistia	0.02	0.01	—	—	—
Mesorhizobium	0.02	0.07	—	—	—
Balneimonas	0.02	0.04	—	—	—
Sphingomonas	0.02	0.02	—	—	—
Butyricicoccus	0.02	0.01	—	—	—
A17	0.01	0.10	—	—	—
Methylosinus	0.01	0.10	—	—	—
Corynebacterium	0.01	0.01	—	—	—
Actinoallomurus	0.01	0.03	—	—	—
Azorhizobium	0.01	0.02	—	—	—
Alicyclobacillus	0.01	0.14	—	—	—
Turicibacter	0.01	0.29	—	—	—
Gemmata	0.01	0.18	—	—	—
Pedobacter	0.01	0.18	—	—	—

Table S3. The relative abundance of the intestinal fungal genera shared by the Hooded Cranes at different lake groups. The genus with a relative abundance of more than 0.01% are listed. PY: Poyang Lake; SJ: Shengjin Lake; CZ: Caizi Lake; F: fungi.

PY_F vs. SJ_F			SJ_F vs. CZ_F		
Genus	Relative abundance (%)		Genus	Relative abundance (%)	
	PY_F	SJ_F		SJ_F	CZ_F
Otidea	16.71	0.54	*Didymella*	18.07	2.37
Emericellopsis	7.23	6.06	*Fusarium*	7.08	0.63
Pyrenochaetopsis	6.84	0.79	*Cystofilobasidium*	6.91	0.13
Mortierella	4.45	2.75	*Emericellopsis*	6.06	0.09
Acremonium	4.12	0.42	*Karstenula*	4.43	1.20
Leucosporidium	2.15	0.49	*Mortierella*	2.75	0.11
Cystofilobasidium	1.43	6.91	*Penicillium*	2.24	0.07
Epicoccum	1.39	0.91	*Plectosphaerella*	1.55	0.18
Cryptococcus	0.84	0.23	*Cladosporium*	0.92	6.10
Nigrospora	0.84	0.03	*Epicoccum*	0.91	10.01
Talaromyces	0.50	0.04	*Pyrenochaetopsis*	0.79	3.34
Neopestalotiopsis	0.40	0.15	*Otidea*	0.54	1.28
Erythrobasidium	0.32	0.06	*Leucosporidium*	0.49	0.40
Sporobolomyces	0.31	0.35	*Phoma*	0.47	0.01
Fusarium	0.30	7.08	*Acremonium*	0.42	0.42
Dioszegia	0.26	0.07	*Trichoderma*	0.39	0.53
Podospora	0.24	0.76	*Aspergillus*	0.38	0.05
Thelebolus	0.19	0.11	*Sporobolomyces*	0.35	4.20
Cladosporium	0.19	0.92	*Cryptococcus*	0.23	0.17
Saitozyma	0.14	0.13	*Neopestalotiopsis*	0.15	0.64
Collarina	0.14	0.01	*Botrytis*	0.13	0.08
Symmetrospora	0.12	0.02	*Saitozyma*	0.13	0.16
Trichoderma	0.11	0.39	*Leptosphaeria*	0.13	0.13
Periconia	0.10	0.01	*Saccharicola*	0.13	0.01
Plectosphaerella	0.07	1.55	*Bullera*	0.12	3.60
Bullera	0.05	0.12	*Buckleyzyma*	0.10	0.04
Penicillium	0.05	2.24	*Vishniacozyma*	0.08	0.15
Rhodotorula	0.05	0.04	*Dioszegia*	0.07	0.08
Pestalotiopsis	0.04	0.01	*Holtermanniella*	0.07	0.01
Aspergillus	0.02	0.38	*Guehomyces*	0.07	9.59
Septoria	0.01	1.24	*Erythrobasidium*	0.06	0.23
Botrytis	0.01	0.13	*Cystobasidium*	0.06	0.45
—	—	—	*Talaromyces*	0.04	0.07
—	—	—	*Hannaella*	0.04	0.31
—	—	—	*Rhodotorula*	0.04	0.04
—	—	—	*Pseudopithomyces*	0.04	0.04
—	—	—	*Nigrospora*	0.03	0.15

—	—	—	*Itersonilia*	0.03	0.01
—	—	—	*Scolecobasidium*	0.02	0.40
—	—	—	*Bulleromyces*	0.02	0.01
—	—	—	*Symmetrospora*	0.02	0.07
—	—	—	Mycosphaerella	0.01	0.03
—	—	—	*Periconia*	0.01	0.03
—	—	—	*Pestalotiopsis*	0.01	0.24
